# Monocyte Proteomics Reveals Involvement of Phosphorylated HSP27 in the Pathogenesis of Osteoporosis

**DOI:** 10.1155/2015/196589

**Published:** 2015-05-07

**Authors:** Bhavna Daswani, Manoj Kumar Gupta, Shubhangi Gavali, Meena Desai, Gajanan J. Sathe, Anushree Patil, Priyanka Parte, Ravi Sirdeshmukh, M. Ikram Khatkhatay

**Affiliations:** ^1^National Institute for Research in Reproductive Health (ICMR), J. M. Street, Parel, Mumbai 400012, India; ^2^Institute of Bioinformatics, International Tech Park, Bangalore 560066, India; ^3^Mazumdar Shaw Centre for Translational Research, Narayana Health, Bangalore 560099, India

## Abstract

Peripheral monocytes, precursors of osteoclasts, have emerged as important candidates for identifying proteins relevant to osteoporosis, a condition characterized by low Bone Mineral Density (BMD) and increased susceptibility for fractures. We employed 4-plex iTRAQ (isobaric tags for relative and absolute quantification) coupled with LC-MS/MS (liquid chromatography coupled with tandem mass spectrometry) to identify differentially expressed monocyte proteins from premenopausal and postmenopausal women with low versus high BMD. Of 1801 proteins identified, 45 were differentially abundant in low versus high BMD, with heat shock protein 27 (HSP27) distinctly upregulated in low BMD condition in both premenopausal and postmenopausal categories. Validation in individual samples (*n* = 80) using intracellular ELISA confirmed that total HSP27 (tHSP27) as well as phosphorylated HSP27 (pHSP27) was elevated in low BMD condition in both categories (*P* < 0.05). Further, using transwell assays, pHSP27, when placed in the upper chamber, could increase monocyte migration (*P* < 0.0001) and this was additive in combination with RANKL (receptor activator of NF_k_B ligand) placed in the lower chamber (*P* = 0.05). Effect of pHSP27 in monocyte migration towards bone milieu can result in increased osteoclast formation and thus contribute to pathogenesis of osteoporosis. Overall, this study reveals for the first time a novel link between monocyte HSP27 and BMD.

## 1. Introduction

Osteoporosis is a potentially crippling condition characterized by low Bone Mineral Density (BMD), micro architectural deterioration of bone tissue, and increased risk of fragility fracture. It is mostly prevalent in postmenopausal women and is only second to cardiovascular disease as a global healthcare concern [1]. Under normal conditions, the skeleton is metabolically active and undergoes continuous remodeling. The key players in bone remodeling are osteoblasts which are responsible for bone formation and osteoclasts which are in charge of bone resorption. An imbalance between the activities of these two cells is the underlying cause for osteoporosis. This imbalance stems from increased bone resorption compared with formation. It has been shown that the number of osteoclasts is inversely correlated with BMD [2], and thus increased osteoclast formation can lead to decreased bone density. Peripheral monocytes are precursors of osteoclasts which migrate towards the bone to differentiate into osteoclasts [3–5]. Hence, proteins relevant to BMD can be unravelled in monocytes which may have important implications in pathogenesis of osteoporosis. Also, circulating monocytes are more easily accessible than osteoclasts which inhabit deep areas of bone cavities, making them better candidates for proteomic studies.

Recently, few elegant studies have shown the comparison of monocyte proteins from women with low versus high BMD in either premenopausal or postmenopausal category [6–8]. The investigators applied two-dimensional electrophoresis or label-free liquid chromatography coupled with tandem mass spectrometry (LC-MS/MS) to identify differentially expressed monocyte proteins in Chinese or Caucasian populations with varying results [6–8]. It is well known that osteoporosis has a strong genetic component and genetic disparities can result in proteomic variations within different ethnic groups [9, 10]. Even though India is one of the largest affected countries [11], studies on proteome patterns in osteoporosis have not been reported in Indian women. Further, BMD measurement by Dual Energy X-ray Absorptiometry (DXA) is gold standard; however, it is site specific. BMD is usually measured at hip and at spine since these are the two sites susceptible to fragility fractures. In fact, BMD at hip and at spine are often discordant (possibly 4 out of every 10 cases) and both sites should be considered for diagnosis of osteoporosis [12–14]. Yet, only BMD at hip was taken into consideration in previous studies on monocyte proteomics in osteoporosis [6–8]. Furthermore, for identification of monocyte proteins solely relevant to BMD and not affected by menopausal status, it may be imperative to study proteins from premenopausal as well as postmenopausal women with low versus high BMD in a single quantitative platform. For this, 4-plex iTRAQ (isobaric Tags for Relative and Absolute Quantification) is best suited to study four different samples in one single experiment with high quantification accuracy [15].

Therefore, our aim was to compare monocyte proteins in low versus high BMD (at hip and at spine) and to identify differentially abundant proteins common to both premenopausal and postmenopausal Indian women, with the purpose of finding proteins relevant to BMD independent of menopausal status, using iTRAQ based LC-MS/MS technology.

## 2. Methods

### 2.1. Study Participants and BMD Measurements

This study was approved by NIRRH Ethics Committee for Clinical Studies. All participants were from the state of Maharashtra, India, and provided their written informed consents for participation. Premenopausal women (*n* = 100) in the age group of 30 to 40 years, with regular menstrual cycles, and postmenopausal women (*n* = 100), aged 50 to 60 years, who had achieved menopause at least one year priorly, were consecutively enrolled. Inclusion criteria comprised women who were apparently healthy, physically active, nonsmokers, nonconsumers of alcohol or any drugs, nonobese, normotensive, and with no nutritional deficiencies as per their weekly dietary recall. Exclusion criteria were chronic disorders of vital organs and metabolic diseases such as diabetes, hypo- and hyperparathyroidism, polycystic ovary syndrome, and autoimmune diseases. Also, women with recent fever or viral infections, taking nutritional supplements, oral contraceptives, or estrogen replacement therapy, were excluded from the study. The enrolled participants underwent Dual Energy X-ray Absorptiometry (DXA, QRR 1000, Hologic Inc., USA) to measure BMD (g/cm^2^) at hip (femoral neck) and at spine (lumbar vertebrae 1–4) at a commercial radiology clinic. *T* score which is used for diagnosis of osteoporosis or osteopenia (a stage prior to osteoporosis) compares individual's BMD values with the expected BMD values of a sex matched healthy young adult. As per World Health Organization criteria, participants were categorized as osteoporotic (*T* score ≤ −2.5 at either of the two sites); osteopenic (*T* score between −1 and −2.5 at either of the two sites); and normal (*T* score ≥ −1 at both sites) [16].

Based on centile distribution, from 100 premenopausal and 100 postmenopausal women, a total of 80 women (20 women belonging to each of the 4 categories, namely, premenopause low BMD, premenopause high BMD, postmenopause low BMD, and postmenopause high BMD) were selected (on the basis of BMD at hip and at spine) for the collection of blood samples. Out of 20 samples in each of the 4 categories, 10 samples were subjected to iTRAQ based quantification (iTRAQ group) and the rest of the samples were stored at −80°C for further use (validation group). It may be noted that all the 20 samples in each of the 4 categories were individually subjected to validation experiments. Baseline characteristics and BMD measurements of the participants have been shown in Table 1. BMD (and *T* scores) at hip and BMD at spine were significantly different in the low versus high BMD groups (*P* < 0.0001), while participants were age and BMI (Body Mass Index) matched in these groups (Table 1).

### 2.2. Monocyte Isolation and Protein Extraction

**Figure 1:**
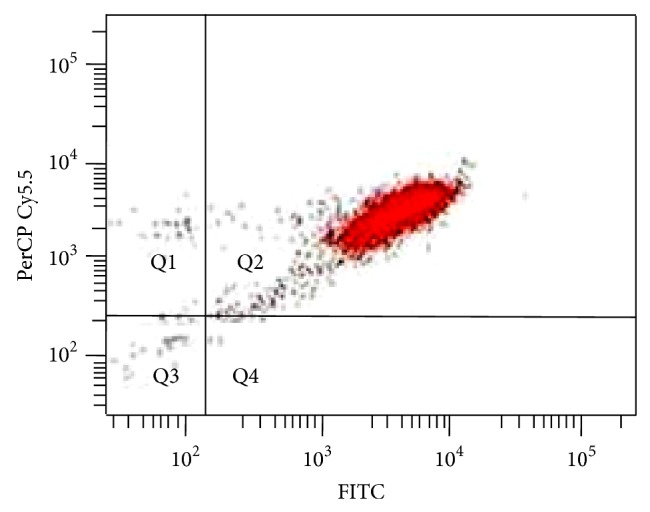
Representative flow cytometry image of isolated monocytes showing ~95% purity. Antibodies against CD14 conjugated to FITC and CD45 conjugated to PerCP Cy5.5 were used for flow cytometry.

Blood was collected from the selected participants (*n* = 80) within three to six months from BMD measurement. Monocyte isolation was carried out by density gradient centrifugation using Histopaque 1077 (Sigma Aldrich, USA) followed by negative immunomagnetic separation using Dynal Monocyte Negative Isolation Kit (Invitrogen, USA). The average purity of monocytes isolated was 95% as confirmed by flow cytometry using antibodies against CD14 conjugated to FITC and CD45 conjugated to PerCP Cy5.5 (Figure 1). For protein extraction, isolated monocytes were resuspended in 0.5% sodium dodecyl sulfate, sonicated, and centrifuged at 14000 g for 30 minutes and the supernatant was collected and stored at −80°C. Protein estimation was performed using Pierce BCA Protein Assay Kit (Thermo Scientific, USA). In each of the 4 categories, equal amounts of protein from 10 samples were pooled, which were subjected to iTRAQ based LC-MS/MS analysis (iTRAQ group), and the remaining 10 samples were stored at −80°C for further use (validation group).

### 2.3. iTRAQ Labelling and Strong Cation Exchange (SCX) Fractionation

Pooled protein (100 *μ*g) from each of the 4 categories was subjected to reduction using tris(2-carboxyethyl) phosphine (TCEP) and cysteine block using methyl methanethiosulfonate (MMTS) followed by trypsin digestion at 37°C for 16 hours using 1 : 20 (w/w) sequencing grade trypsin (Promega, USA). The tryptic digests were labelled with 4 different iTRAQ reagents: premenopause low BMD with 114, premenopause high BMD with 115, postmenopause low BMD with 116, and postmenopause high BMD with 117 according to the manufacturer's instructions (Applied Biosystems, USA). The peptides with the four labels were pooled, dried, and reconstituted in solvent A (5 mM KH_2_PO_4_ pH 2.7, 30% ACN) and subjected to fractionation by a SCX column (PolySULFOETHYL A column, 100 × 2.1 mm, 5 *μ*m particles with 300 Å pores; PolyLC, Columbia, MD) using an Agilent 1200 series LC system. A gradient of 50 min from 5 to 40% solvent B (350 mM KCl in solvent A) with flow rate of 300 *μ*L/min was applied. A total of 15 SCX fractions were desalted using C_18_ microtips, vacuum-dried, and stored at −80°C until mass spectrometric analysis. The sample labelling and fractionation were performed twice using the same pool of samples to generate an experimental replicate.

### 2.4. LC-MS/MS

Thus, 30 desalted SCX fractions were reconstituted in 20 *μ*L of 0.1% formic acid and subjected to LC-MS/MS analysis using LTQ Orbitrap Velos mass spectrometer (Thermo Fischer Scientific, Bremen, Germany) coupled with Proxeon Easy nLC system (Thermo Scientific, Bremen, Germany) as performed previously [17]. In brief, magic C18 AQ reversed phase material (Michrom Bioresources, 5 *μ*m, 100 Å) was used to make in house chromatographic capillary columns. Trap column (75 *μ*m × 2 cm) at a flow rate of 3 *μ*L/min was used to enrich the peptides followed by analytical column (75 *μ*m × 10 cm), at a flow rate of 350 nL/min, which was used to resolve the peptides. A linear gradient of 7–30% ACN was used for 60 min to elute the peptides from the analytical column. Data was acquired in a data dependent mode at a mass resolution of 60,000 at 400* m*/*z*. For fragmentation, the activation method used was high-energy collision dissociation (HCD) at 41% collision energy with a dynamic exclusion window of 45 seconds [17]. Top twenty peptides with the most intense peaks were selected for each duty cycle and detected at a mass resolution of 15,000 at 400* m/z*. The automatic gain control for full FT MS was million ions and for FT MS/MS was set to 0.1 million ions with a maximum time of accumulation of 250 milliseconds. To increase accuracy of mass measurements, the “lock mass” option was used.

### 2.5. Protein Identification and Relative Quantification

The raw files generated by the mass spectrometer were analyzed using Proteome Discoverer software version 1.3 (Thermo Scientific, Bremen, Germany) using Sequest as the search algorithm. Searches were made against NCBI human RefSeq database (release 52) containing 33,985 proteins. The settings for the search parameters were the same as used previously [17]. In brief, the settings for the search included selection of enzyme used as trypsin with one missed cleavage allowed and oxidation of methionine and alkylation at cysteine as dynamic and static modification, respectively, and iTRAQ modification at N-terminus of the peptide and at lysine. The tolerance for precursor mass was 20 ppm and fragment mass was 0.1 Da. High peptide tolerance and top one peptide rank filters were applied. False discovery rate was set at 1% (strict) at peptide level to increase confidence of peptide identifications. Differentially abundant proteins (≥1.5-fold) common to both replicate experiments were listed. Bioinformatics analysis was carried out to categorize proteins based on biological processes, cellular component, and molecular function classification using annotations in Human Protein Reference Database (HPRD, http://hprd.org/) [18], which is in compliance with gene ontology (GO) standards.

### 2.6. ELISA

HSP27 was estimated in 80 subjects' monocyte protein samples (20 samples in each of the 4 groups) using human total HSP27 intracellular ELISA kit (R&D systems, USA) and Phospho-HSP27 (S78/S82) intracellular ELISA kit (R&D systems, USA). Phosphorylation at serine 78 and/or serine 82 was estimated as these two sites are the major sites for HSP27 phosphorylation [19]. For total HSP27, 1 *μ*g/mL goat anti-human antibody in phosphate buffered saline (PBS) was adsorbed on a 96-well plate overnight. After blocking for 1 hour with 1% bovine serum albumin (BSA), 100 *μ*L samples (1 *μ*g monocyte protein) or standards (range = 7.8–8000 pg/mL) in duplicate were incubated for 2 hours. Biotinylated rabbit anti-human HSP27 was added at a concentration of 0.1 *μ*g/mL for 2 hours followed by washing and addition of streptavidin conjugated to horseradish peroxidase for 2 hours, tetramethylbenzidine substrate for 20 min, and 2N sulphuric acid stop solution. The plate was read at 450 nm and for correction at 540 nm. Likewise, phosphorylated HSP27 was estimated in monocyte protein samples using the same procedure as above except with phosphospecific standards and antibodies, with the concentration of capture antibody being 2 *μ*g/mL and detection antibody being 500 ng/mL. HSP70 cross-reactivity as provided by the manufacturer was 0.23% for total protein and 0% for phosphorylated protein. Phosphorylated protein was calculated for each individual sample and expressed as percentage (ratio of phosphorylated protein to total protein multiplied by 100) [20]. This normalization represents the real change in phosphorylation which is not due to mere change in level of expression [20].

### 2.7. Monocyte Migration Assay

The transwell migration of human monocytes (PromoCell, USA) was studied in 24-well cell culture plates with polycarbonate membrane inserts having pores size of 5 *μ*m (Corning, USA). The media used were phenol red free RPMI 1640 (Gibco, USA). The upper chamber of all wells contained a final volume of 200 *μ*L medium containing 0.5% BSA, Sigma, USA, wherein 1 × 10^5^ cells were suspended. The lower chambers of all wells contained a final volume of 600 *μ*L medium containing 10 ng/mL of monocyte chemoattractant protein-1 (MCP-1) (Abcam, USA) (except negative controls which contained only medium). Increasing concentrations (0.1 ng/mL, 1 ng/mL, 10 ng/mL, 100 ng/mL, and 1000 ng/mL) of recombinant phosphorylated HSP27 (Enzo Life Sciences) were placed in either upper or lower chambers in separate experiments. We also compared phosphorylated HSP27 with receptor activator of NF_k_B ligand (RANKL), an osteoclast factor known to attract monocytes. For this, recombinant human RANKL (100 ng/mL, Invitrogen, USA) was placed in lower chambers and phosphorylated HSP27 (10 ng/mL) was placed in either upper or lower chambers. Migration was allowed to proceed for two and half hours at 37°C in 5% CO_2_. The migrated cells suspended in the lower chamber were counted using a haemocytometer. All assays were performed thrice in triplicate.

### 2.8. Statistical Analysis

Data is represented as mean ± standard error of mean (SEM). GraphPad Prism software version 6 was used for Student's *t*-test, ANOVA, and graphical representations. Comparisons of age, BMI, BMD, *T* scores, and HSP27 between low and high BMD groups were performed using Student's *t*-test. Multivariate logistic regression was applied to determine odds ratio for HSP27 to differentiate between low and high BMD using Statistical Package for Social Sciences (SPSS) Windows version 16. For migration assays, one way ANOVA (analysis of variance) with post hoc test for linear trend was employed. Treatment with RANKL and phosphorylated HSP27 versus RANKL alone was compared using Student's *t*-test. *P* value ≤ 0.05 was considered statistically significant.

## 3. Results 

### 3.1. Protein Identifications and Differentially Expressed Proteins

The spectra generated by tandem mass spectrometry revealed 30,608 peptides and 1801 proteins (including CD14) which were common to both replicate experiments. The experimental replicates revealed 45 differentially abundant proteins (≥1.5-fold) in low versus high BMD condition in either premenopausal (pre) or postmenopausal (post) women. The differential proteins and their fold change and peptides and gene ontology information are shown in Supporting Information Table 1 (see Supplementary Material available online at http://dx.doi.org/10.1155/2015/196589).

These proteins belonged to various biological processes such as protein metabolism (HSPB1, CCT4, RPS12, and RPS28); cell communication/signal transduction (RAC1, RHOT2, ANXA1, ANXA2, ANXA6, CARHSP1, and EEF1D); cell growth and/or maintenance (VIM, CAPG, TUBB6, and FMNL1); immune response (SAMHD1, HLA-DR, DEFA1, and ARMCX3); metabolism/energy pathways (LYZ, PRMT1, and SULT1A1); regulation of nucleic acid metabolism (HIST1H4I, HIST1H1B, HIST1H2BB, HNRNPC, HNRNPD, HNRNPU, HNRNPUL2, RNASE2, SET, XRCC5, MNDA, KHDRBS1, and DCLRE1A); transport (SLC2A3, VPS37A, ABCB6, and KDELR1); apoptosis (PYCARD); and unknown processes (CPPED1, SPRYD4, TMED3, and YIF1B).

In order to identify differentially abundant proteins commonly regulated in pre and post categories, they were categorized as “concordant” (same protein up/downregulated in both categories), “discordant” (same protein upregulated in one category and downregulated in another category), and “dissimilar” (different proteins up/downregulated in both categories) shown in Table 2.

**Figure 2:**
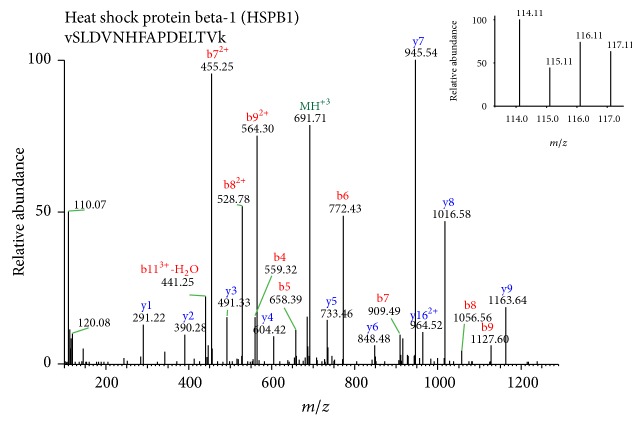
Representative MS/MS spectra with reporter ions showing differential abundance of HSPB1. Monocyte proteins from premenopausal (pre) women and postmenopausal (post) women with low versus high BMD were labelled with iTRAQ reagents 114 (pre low), 115 (pre high), 116 (post low), and 117 (post high).

As mentioned earlier, our aim was to detect BMD based protein alteration concordant in both categories. In this regard, it was interesting to find that HSPB1 protein (heat shock protein 27 or HSP27) was upregulated in low BMD condition in both pre and post categories. Representative MS/MS spectra are shown in Figure 2. HSP27 is a molecular chaperone belonging to the small HSP group. Despite the fact that HSP27 has gained prominence in recent times [21], its relevance to BMD is currently unknown. Therefore, HSP27 was selected for validation in individual samples (*n* = 80). In addition, it is known that its phosphorylated form is a multifunctional protein with roles beyond chaperone activity including promigratory activities [21]. Hence, we also assessed levels of phosphorylated HSP27 in all the individual samples (*n* = 80) and its functional relevance in transwell migration assays was assessed.

### 3.2. Total HSP27 or tHSP27

**Figure 3:**
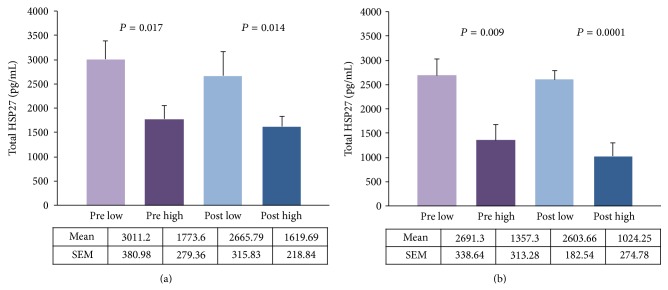
Total HSP27 in monocytes. Premenopausal (pre) and postmenopausal (post) women with low versus high BMD: (a) iTRAQ group, (b) validation group.

Levels of tHSP27 were significantly increased in the low BMD groups as compared to the high BMD groups in both pre (*P* = 0.017 for iTRAQ group and *P* = 0.009 for validation group) and post (*P* = 0.014 for iTRAQ group and *P* = 0.0001 for validation group) categories as shown in Figure 3.

### 3.3. Phosphorylated HSP27 or pHSP27

**Figure 4:**
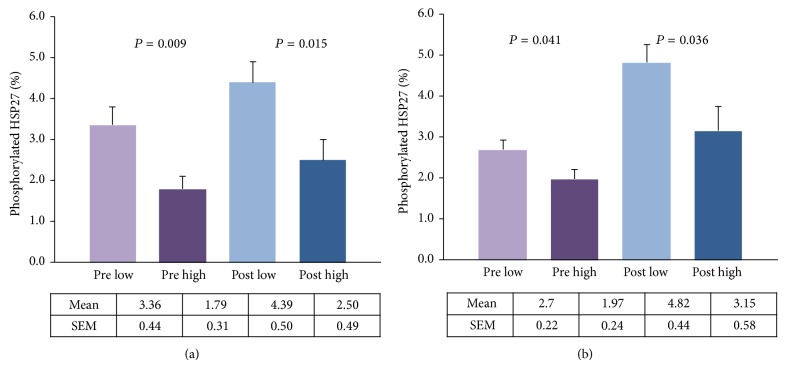
Phosphorylated HSP27 in monocytes. Premenopausal (pre) and postmenopausal (post) women with low versus high BMD: (a) iTRAQ group, (b) validation group.

Levels of pHSP27 were also significantly elevated in low BMD groups compared to high BMD groups in both pre (*P* = 0.009 for iTRAQ group and *P* = 0.041 for validation group) and post (*P* = 0.015 for iTRAQ group and *P* = 0.036 for validation group) categories as shown in Figure 4.

### 3.4. Predictive Ability of Monocyte tHSP27 and pHSP27

In pre category, odds ratio to distinguish between low and high BMD was 1.001 (confidence interval (CI) = 1.001–1.002, *P* = 0.011) for tHSP27 and was 3.826 (CI = 1.226–11.620, *P* = 0.017) for pHSP27. In post category, odds ratio to distinguish between low and high BMD was 1.003 (CI = 1.001–1.004, *P* = 0.003) for tHSP27 and was 2.763 (CI = 1.231–6.202, *P* = 0.014) for pHSP27. Thus, pHSP27 was a better indicator of low BMD compared to tHSP27, in both pre and post categories.

### 3.5. Effect of Phosphorylated HSP27 on Monocyte Transwell Migration

**Figure 5:**
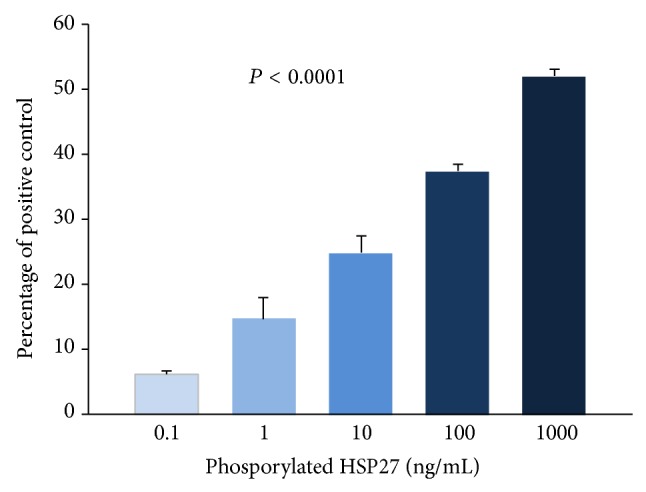
Effect of phosphorylated HSP27 (pHSP27) on monocyte transwell migration. Migration is represented as percentage of positive control (MCP-1; 10 ng/mL in the lower chambers). Increasing concentrations (ng/mL) of pHSP27 were placed in the upper chambers with MCP-1 (10 ng/mL) in the lower chambers.

**Figure 6:**
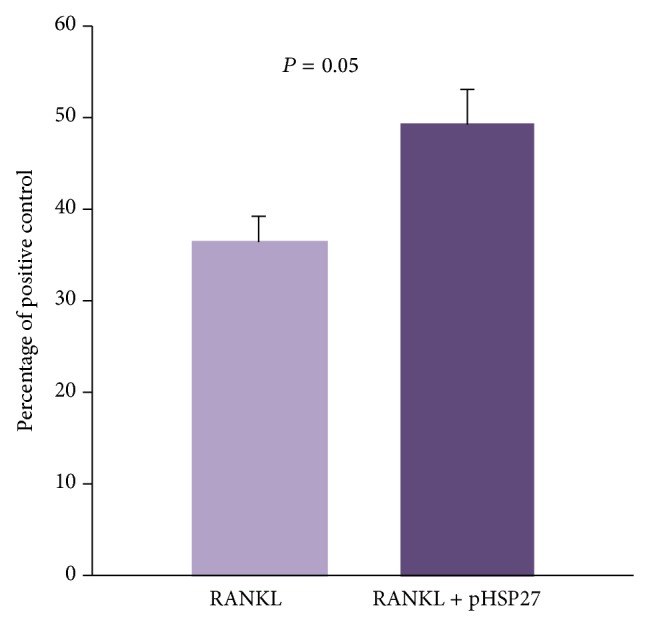
Monocyte transwell migration towards RANKL with and without phosphorylated HSP27 (pHSP27). Migration is represented as percentage of positive control (MCP-1; 10 ng/mL in the lower chambers). In the lower chamber, RANKL (100 ng/mL) was placed with or without pHSP27 (10 ng/mL) in the upper chamber.

On addition of increasing concentrations of recombinant phosphorylated HSP27 in the upper chamber, there was a significant dose dependent increase in monocyte migration (*P* < 0.0001) shown in Figure 5. However, when placed in the lower chamber, increasing concentrations of recombinant phosphorylated HSP27 showed no significant change in migration (data not shown). Further, recombinant RANKL was placed in the lower chambers with or without recombinant phosphorylated HSP27 placed in the upper chamber. Monocyte migration increased with the presence of phosphorylated HSP27 along with RANKL compared to RANKL alone (*P* = 0.05) shown in Figure 6.

## 4. Discussion

In the present study, monocyte proteomes from women with low versus high BMD were investigated for the first time in a single iTRAQ based quantitative platform with the purpose of identifying BMD based differentially expressed proteins irrespective of menopausal status. Using high throughput proteomics technology, 1801 monocyte proteins were identified, out of which 45 proteins were differentially abundant in low versus high BMD condition in premenopausal and/or postmenopausal women. Protein alterations in low versus high BMD condition were different in premenopausal and postmenopausal categories and this is expected due to differences in age and hormonal milieu. Notably, heat shock protein 27 (HSP27) was upregulated in monocytes in low BMD compared with high BMD condition independent of menopausal status. This was confirmed using intracellular ELISA for total HSP27 (tHSP27) as well as phosphorylated HSP27 (pHSP27) in iTRAQ samples as well as additional samples. Interestingly, pHSP27 showed a superior ability compared to tHSP27 for distinguishing between low and high BMD. We further examined whether phosphorylated form of HSP27 has a functional role that can contribute to low BMD. Indeed, we found that recombinant phosphorylated HSP27 (rpHSP27) increased monocyte migration in a dose dependent manner and had an additive effect in combination with the chemoattraction by recombinant RANKL (rRANKL).

The nonphosphorylated form of HSP27 is primarily a molecular chaperone, whereas phosphorylation turns on many new activities making it a multifunctional moonlighting protein [21]. Strikingly, pHSP27 has gained prominence in recent times due to its distinctive role in inducing actin reorganization and increasing cell migration. This phenomenon has been demonstrated in neutrophils, smooth muscle cells, and endothelial cells amongst other cell types [22–25]. Despite this, till date, its effect on monocyte migration had not been studied. HSP27 also exists as a secreted protein and its exogenous form can exert its effects by entering into the cells [26]. Hence, we studied the effect of exogenous addition of rpHSP27 on monocyte transwell migration. Interestingly, rpHSP27 significantly increased migration of monocytes in a dose dependent manner when added to the upper chambers and this change in migration was not seen when rpHSP27 was added to the lower chambers. This is likely since rpHSP27 is known to induce actin reorganization thereby increasing the motility of the cells; however, it may not function as a chemoattractant by itself. More importantly, we observed that rpHSP27 could boost recombinant rRANKL mediated chemoattraction of monocytes. RANKL is a vital osteoclast maturating factor [3]. Its secreted form is abundantly present in the vicinity of the skeleton and acts as a powerful chemoattractant for monocyte migration towards bone [27, 28]. Hence, migration towards rRANKL represents migration towards the bone milieu, which constitutes the initial stage of osteoclast formation. Therefore, pHSP27 is involved in the early stage of osteoclastogenesis which can contribute to pathogenesis of osteoporosis.

In addition, it is proven that pHSP27 can inhibit stress induced cell death [29]. Osteoclast formation involves reactive oxygen species [30] and hence antiapoptotic activity of pHSP27 may foster monocyte survival in such conditions and thus further augment osteoclastogenesis.

Oxidative stress, a typical feature of low BMD, may be responsible for the initial upregulation of total HSP27 in low BMD condition [31]. The concomitant rise in phosphorylation relative to total HSP27 is plausibly due to proinflammatory cytokines like interleukin-1, interleukin-6, and tumor necrosis factor-alpha, which are known to be elevated in low BMD condition and can stimulate phosphorylation of HSP27 [32–35]. Further, HSP27 expression may rise in many stress related conditions; even so, its novel relevance to bone physiology can be deduced from our study owing to the strict selection criteria of cases and controls.

In summary, our results bring to light an inverse association between monocyte HSP27 protein and bone density in Indian women. The increased phosphorylation of HSPB1 protein may contribute to osteoclastogenesis through increase of monocyte lifespan and increase in migration of monocytes towards the bone microenvironment eventually leading to low BMD.

## 5. Conclusions

The quest for newer insights into the complex pathogenetic mechanisms of osteoporosis has revealed new candidate molecules in monocytes previously unknown. We identified and validated a novel link between BMD and HSP27 in monocytes in both premenopausal and postmenopausal Indian women. It can be inferred that level of HSP27 phosphorylation in monocytes may be an important determinant of BMD in Indian women and may serve as an early predictive indicator of low BMD independent of menopausal status. Moreover, pHSP27 can increase monocyte migration towards the bone milieu which may play a pivotal role in pathogenesis of osteoporosis. Further research needs to be steered towards understanding the role of pHSP27 on monocyte commitment and differentiation to the osteoclast lineage.

## Supplementary Material

The differential proteins and their fold change, peptides and gene ontology information are shown in Supporting Information Table 1

## Figures and Tables

**Table 1:** Baseline traits and BMD measurements of participants (iTRAQ and validation groups).

Parameter	Premenopausal women			Postmenopausal women		
	Low BMD (*n* = 10)	High BMD (*n* = 10)	*P* value	Low BMD (*n* = 10)	High BMD (*n* = 10)	*P* value
iTRAQ group						
Age (years)	36.1 ± 1.2	36 ± 1.1	ns	55.7 ± 1.1	53.8 ± 0.8	ns
BMI (Kg/m^2^)	24.7 ± 0.9	26.3 ± 0.6	ns	26.3 ± 0.8	28.5 ± 1.1	ns
BMD at hip (g/cm^2^)	0.796 ± 0.02	1.07 ± 0.01	<0.0001	0.699 ± 0.02	1.008 ± 0.02	<0.0001
BMD at spine (g/cm^2^)	0.980 ± 0.05	1.247 ± 0.03	<0.0001	0.796 ± 0.03	1.154 ± 0.02	<0.0001
*T* score hip	−1.5 ± 0.13	0.76 ± 0.09	<0.0001	−2.33 ± 0.14	0.23 ± 0.17	<0.0001
*T* score spine	−1.66 ± 0.12	0.57 ± 0.24	<0.0001	−3.2 ± 0.22	−0.16 ± 0.18	<0.0001
OPP/OP/N	OP = 10	*N* = 10	—	OPP = 9, OP = 1	*N* = 10	—

Validation group						
Age (years)	35.7 ± 0.8	34.8 ± 1.2	ns	54.2 ± 0.9	53.6 ± 0.9	ns
BMI (Kg/m^2^)	23.8 ± 0.9	25.6 ± 1.2	ns	24.9 ± 0.9	25.3 ± 0.5	ns
BMD at hip (g/cm^2^)	0.805 ± 0.01	1.075 ± 0.02	<0.0001	0.707 ± 0.02	1.053 ± 0.02	<0.0001
BMD at spine (g/cm^2^)	1.063 ± 0.04	1.219 ± 0.03	0.0029	0.840 ± 0.02	1.189 ± 0.03	<0.0001
*T* score hip	−1.5 ± 0.11	0.79 ± 0.16	<0.0001	−2.21 ± 0.22	0.61 ± 0.15	<0.0001
*T* score spine	−0.97 ± 0.24	0.31 ± 0.23	0.0033	−2.84 ± 0.20	0.08 ± 0.23	<0.0001
OPP/OP/N	OP = 10	*N* = 10	—	OPP = 8, OP = 2	*N* = 10	—

Values are mean ± SEM, ns is not significant, BMD is Bone Mineral Density, BMI is Body Mass Index, OPP stands for osteoporosis, OP stands for osteopenia, and N stands for normal.

**Table 2:** 45 differentially abundant proteins in low BMD condition.

Regulation	Premenopausal women	Postmenopausal women
Concordant	HSPB1↑	HSPB1↑

Discordant	RHOT2↓, YIF1B↓, ABCB6↓, DEFA1↑, SULT1A1↑	RHOT2↑, YIF1B↑, ABCB6↑, DEFA1↓, SULT1A1↓

Dissimilar	RAC1↓, ARMCX3↓, RPS28↓, PRMT1↑, DCLRE1A↓, VPS37A↑, SLC2A3↓, TMED3↓, CCT4↑	ANXA1↓, ANXA2↓, ANXA6↓, LYZ↓, TUBB6↑, HIST1H4I↓, HIST1H1B↓, HIST1H2BD↓, HIST1H2BB↓, SET↓, HNRNPU↓, HNRNPUL2↓, HNRNPD↓, HNRNPC↓, XRCC5↓, VIM↓, MNDA↑, RNASE2↓, KDELR1↑, KHDRBS1↓, RPS12↓, PYCARD↓, SAMHD1↓, HLA-DRA↓, CARHSP1↑, CAPG↓, FMNL1↓, SPRYD4↑, CPPED1↓, EEF1D↓

## References

[1] International Osteoporosis Foundation (2002). *Osteoporosis in the European Community: A Call for Action*.

[2] D'Amelio P., Grimaldi A., Pescarmona G. P., Tamone C., Roato I., Isaia G. (2005). Spontaneous osteoclast formation from peripheral blood mononuclear cells in postmenopausal osteoporosis. *The FASEB Journal*.

[3] Massey H. M., Flanagan A. M. (1999). Human osteoclasts derive from CD14-positive monocytes. *British Journal of Haematology*.

[4] Nicholson G. C., Malakellis M., Collier F. M. (2000). Induction of osteoclasts from CD14-positive human peripheral blood mononuclear cells by receptor activator of nuclear factor *κ*B ligand (RANKL). *Clinical Science*.

[5] Kotani M., Kikuta J., Klauschen F. (2013). Systemic circulation and bone recruitment of osteoclast precursors tracked by using fluorescent imaging techniques. *Journal of Immunology*.

[6] Deng F.-Y., Liu Y.-Z., Li L.-M. (2008). Proteomic analysis of circulating monocytes in Chinese premenopausal females with extremely discordant bone mineral density. *Proteomics*.

[7] Deng F.-Y., Lei S.-F., Zhang Y. (2011). Peripheral blood monocyte-expressed *ANXA2* gene is involved in pathogenesis of osteoporosis in humans.. *Molecular & Cellular Proteomics*.

[8] Deng F. Y., Zhu W., Zeng Y. (2014). Is GSN significant for hip BMD in female Caucasians?. *Bone*.

[9] García-Bailo B., Brenner D. R., Nielsen D. (2012). Dietary patterns and ethnicity are associated with distinct plasma proteomic groups. *The American Journal of Clinical Nutrition*.

[10] Kim C. X., Bailey K. R., Klee G. G. (2010). Sex and ethnic differences in 47 candidate proteomic markers of cardiovascular disease: the Mayo Clinic proteomic markers of arteriosclerosis study. *PLoS ONE*.

[11] Osteoporosis Society of India (2003). *Action Plan Osteoporosis: Consensus statement of an Expert Group*.

[12] O'Gradaigh D., Debiram I., Love S., Richards H. K., Compston J. E. (2003). A prospective study of discordance in diagnosis of osteoporosis using spine and proximal femur bone densitometry. *Osteoporosis International*.

[13] Mounach A., Mouinga Abayi D. A., Ghazi M. (2009). Discordance between hip and spine bone mineral density measurement using DXA: prevalence and risk factors. *Seminars in Arthritis and Rheumatism*.

[14] Singh M., Magon N., Singh T. (2012). Major and minor discordance in the diagnosis of postmenopausal osteoporosis among Indian women using hip and spine dual-energy X-ray absorptiometry. *Journal of Mid-Life Health*.

[15] Li Z., Adams R. M., Chourey K., Hurst G. B., Hettich R. L., Pan C. (2012). Systematic comparison of label-free, metabolic labeling, and isobaric chemical labeling for quantitative proteomics on LTQ orbitrap velos. *Journal of Proteome Research*.

[16] (1993). Consensus development conference: diagnosis, prophylaxis, and treatment of osteoporosis. *The American Journal of Medicine*.

[17] Polisetty R. V., Gautam P., Sharma R. (2012). LC-MS/MS analysis of differentially expressed glioblastoma membrane proteome reveals altered calcium signaling and other protein groups of regulatory functions. *Molecular & Cellular Proteomics*.

[18] Keshava Prasad T. S., Goel R., Kandasamy K. (2009). Human protein reference database—009 update. *Nucleic Acids Research*.

[19] Landry J., Lambert H., Zhou M. (1992). Human HSP27 is phosphorylated at serines 78 and 82 by heat shock and mitogen-activated kinases that recognize the same amino acid motif as S6 kinase II. *The Journal of Biological Chemistry*.

[20] Wu R., Dephoure N., Haas W. (2011). Correct interpretation of comprehensive phosphorylation dynamics requires normalization by protein expression changes. *Molecular & Cellular Proteomics*.

[21] Vidyasagar A., Wilson N. A., Djamali A. (2012). Heat shock protein 27 (HSP27): biomarker of disease and therapeutic target. *Fibrogenesis & Tissue Repair*.

[22] Jog N. R., Jala V. R., Ward R. A., Rane M. J., Haribabu B., McLeish K. R. (2007). Heat shock protein 27 regulates neutrophil chemotaxis and exocytosis through two independent mechanisms. *Journal of Immunology*.

[23] Hedges J. C., Dechert M. A., Yamboliev I. A. (1999). A role for p38^MAPK^/HSP27 pathway in smooth muscle cell migration. *The Journal of Biological Chemistry*.

[24] Piotrowicz R. S., Hickey E., Levin E. G. (1998). Heat shock protein 27 kDa expression and phosphorylation regulates endothelial cell migration. *The FASEB Journal*.

[25] Shin K. D., Lee M.-Y., Shin D.-S. (2005). Blocking tumor cell migration and invasion with biphenyl isoxazole derivative KRIBB3, a synthetic molecule that inhibits Hsp27 phosphorylation. *The Journal of Biological Chemistry*.

[26] Laudanski K., De A., Miller-Graziano C. (2007). Exogenous heat shock protein 27 uniquely blocks differentiation of monocytes to dendritic cells. *European Journal of Immunology*.

[27] Breuil V., Schmid-Antomarchi H., Schmid-Alliana A., Rezzonico R., Euller-Ziegler L., Rossi B. (2003). The receptor activator of nuclear factor (NF)-kappaB ligand (RANKL) is a new chemotactic factor for human monocytes. *The FASEB Journal*.

[28] Collin-Osdoby P., Rothe L., Anderson F., Nelson M., Maloney W. (2001). Receptor activator of NF-kappa B and osteoprotegerin expression by human microvascular endothelial cells, regulation by inflammatory cytokines, and role in human osteoclastogenesis. *The Journal of Biological Chemistry*.

[29] Charette S. J., Lavoie J. N., Lambert H., Landry J. (2000). Inhibition of Daxx-mediated apoptosis by heat shock protein 27. *Molecular and Cellular Biology*.

[30] Lee N. K., Choi Y. G., Baik J. Y. (2005). A crucial role for reactive oxygen species in RANKL-induced osteoclast differentiation. *Blood*.

[31] Basu S., Michaëlsson K., Olofsson H., Johansson S., Melhus H. (2001). Association between oxidative stress and bone mineral density. *Biochemical and Biophysical Research Communications*.

[32] Zheng S. X., Vrindts Y., Lopez M. (1997). Increase in cytokine production (IL-1*β*, IL-6, TNF-*α* but not IFN-*γ*, GM-CSF or LIF) by stimulated whole blood cells in postmenopausal osteoporosis. *Maturitas*.

[33] Ahlers A., Belka C., Gaestel M. (1994). Interleukin-1-induced intracellular signaling pathways converge in the activation of mitogen-activated protein kinase and mitogen-activated protein kinase-activated protein kinase 2 and the subsequent phosphorylation of the 27-kilodalton heat shock protein in monocytic cells. *Molecular Pharmacology*.

[34] Belka C., Ahlers A., Sott C., Gaestel M., Herrmann F., Brach M. A. (1995). Interleukin (IL)-6 signaling leads to phosphorylation of the small heat shock protein (Hsp)27 through activation of the MAP kinase and MAPKAP kinase 2 pathway in monocytes and monocytic leukemia cells. *Leukemia*.

[35] Mehlen P., Mehlen A., Guillet D., Preville X., Arrigo A.-P. (1995). Tumor necrosis factor-*α* induces changes in the phosphorylation, cellular localization, and oligomerization of human hsp27, a stress protein that confers cellular resistance to this cytokine. *Journal of Cellular Biochemistry*.

